# CD85k Contributes to Regulatory T Cell Function in Chronic Viral Infections

**DOI:** 10.3390/ijms22010031

**Published:** 2020-12-22

**Authors:** Anna Estrada Brull, Felix Rost, Josua Oderbolz, Florian R. Kirchner, Salomé Leibundgut-Landmann, Annette Oxenius, Nicole Joller

**Affiliations:** 1 Institute of Experimental Immunology, University of Zurich, 8057 Zurich, Switzerland; anna.estradabrull@uzh.ch (A.E.B.); rost@immunology.uzh.ch (F.R.); florian.kirchner@uzh.ch (F.R.K.); salome.leibundgut-landmann@uzh.ch (S.L.-L.); 2 ETH Zurich, Institute of Microbiology, 8093 Zurich, Switzerland; josua.oderbolz@micro.biol.ethz.ch (J.O.); aoxenius@micro.biol.ethz.ch (A.O.); 3 Section of Immunology, Vetsuisse Faculty, University of Zurich, 8057 Zurich, Switzerland

**Keywords:** regulatory T cells, chronic viral infection, CD85k, ALCAM

## Abstract

Regulatory T cells (Tregs) prevent excessive immune responses and limit immune pathology upon infections. To fulfill this role in different immune environments elicited by different types of pathogens, Tregs undergo functional specialization into distinct subsets. During acute type 1 immune responses, type 1 Tregs are induced and recruited to the site of ongoing Th1 responses to efficiently control Th1 responses. However, whether a similar specialization process also takes place following chronic infections is still unknown. In this study, we investigated Treg specialization in persistent viral infections using lymphocytic choriomeningitis virus (LCMV) and murine cytomegalovirus (MCMV) infection as models for chronic and latent infections, respectively. We identify CD85k as a Th1-specific co-inhibitory receptor with sustained expression in persistent viral infections and show that recombinant CD85k inhibits LCMV-specific effector T cells. Furthermore, expression of the CD85k ligand ALCAM is induced on LCMV-specific and exhausted T cells during chronic LCMV infection. Finally, we demonstrate that type 1 Tregs arising during chronic LCMV infection suppress Th1 effector cells in an ALCAM-dependent manner. These results extend the current knowledge of Treg specialization from acute to persistent viral infections and reveal an important functional role of CD85k in Treg-mediated suppression of type 1 immunity.

## 1. Introduction

Regulatory T cells (Tregs) are a distinct subset of CD4^+^ T helper cells characterized by the expression of the transcription factor Foxp3 that play an essential role in maintaining immune tolerance and homeostasis [1,2,3]. In addition to inhibiting autoimmune responses, Tregs play a critical role in ensuring controlled immune responses upon pathogen encounter [4]. However, while immune suppression through Tregs limits immune pathology, it may also promote pathogen persistence [5,6,7,8].

Substantial heterogeneity is observed within the Treg pool and ongoing immune responses induce Treg specialization into different subsets depending on the immune environment [9,10,11]. During type 1 immune responses, IFN-γ as well as IL-27 drive the specialization of Tregs into type 1 Tregs, which are essential for effective control of Th1 responses [9,12,13]. Type 1 Tregs co-express Foxp3 and the Th1 master transcription factor T-bet, which in turn induces expression of the chemokine receptor CXCR3 [14]. The mirrored expression of CXCR3 enables type 1 Tregs to migrate to the same inflammatory sites as the effector Th1 cells and control local immune responses efficiently.

In addition to T-bet and CXCR3, type 1 Tregs also express a set of Th1-specific co-inhibitory receptors, including Lag-3 and the novel co-inhibitory receptor CD85k (also known as LILRB4, ILT3, or gp49D), which are predictive of their suppressive function [15]. CD85k was originally reported as a surface receptor expressed on myeloid cells that renders DCs tolerogenic [16,17]. Recombinant CD85k has a similar tolerizing effect and binds to a ligand that is only expressed on T cells upon their activation [18]. This long elusive ligand has recently been identified as ALCAM (or CD166) [19], enabling further functional studies on CD85k. While we and others have found CD85k not only being expressed on tolerogenic DCs but also on Tregs [15,20], it remained unclear whether this molecule functionally contributes to Treg-mediated suppression, particularly in the context of Th1 responses, where we found it to be specifically induced in Tregs [15].

Acute infections eliciting Th1 responses induce the expansion of type 1 Tregs followed by contraction, once the infection is resolved [9,12,13,15]. Whether a similar functional specialization process of Tregs takes place in persistent infections and whether this is maintained as the pathogen persists is still unknown. Persistent infections, in which the virus is not cleared from the individual, can be categorized into chronic infections, in which the virus in continuously produced and latent infections, characterized by the lack of infectious virus except for episodes of reactivation. Patients with chronic HIV or hepatitis A and B virus infection and people latently infected with cytomegalovirus (CMV) harbor elevated Treg frequencies [21,22,23,24], suggesting an active role of Tregs during persistent viral infections. Furthermore, Tregs have been shown to suppress anti-viral responses and contribute to viral persistence in herpes simplex virus, Friend virus, or murine CMV (MCMV) infection [6,7,25,26]. Hence, Tregs play an active role during persistent viral infections, but whether this requires their functional specialization into type 1 Tregs has not been addressed.

In the present study, we examined and characterized Treg responses in persistent viral infections, which elicit Th1 immune responses. We show that Tregs isolated from lymphocytic choriomeningitis virus (LCMV)-infected mice retain an unimpaired suppressive capacity. Furthermore, we describe common phenotypic features of Treg responses in persistent infections and identify CD85k as a co-inhibitory receptor with sustained expression in both chronic LCMV and latent MCMV infection. We show that recombinant CD85k can inhibit LCMV-specific effector T cells and that the CD85k ligand ALCAM is induced on LCMV-specific effector T cells during chronic LCMV infection. We also found ALCAM expression to be maintained on exhausted T cells. Finally, we show that type 1 Tregs arising during chronic LCMV infection suppress Th1 effector cells in an ALCAM-dependent manner. These results extend our current knowledge of Treg specialization from acute to persistent viral infections and reveal an important functional role of CD85k in Treg-mediated control of persistent Th1 responses.

## 2. Results

### 2.1. Tregs Maintain Suppressive Function during Chronic Infection

**Figure 1:**
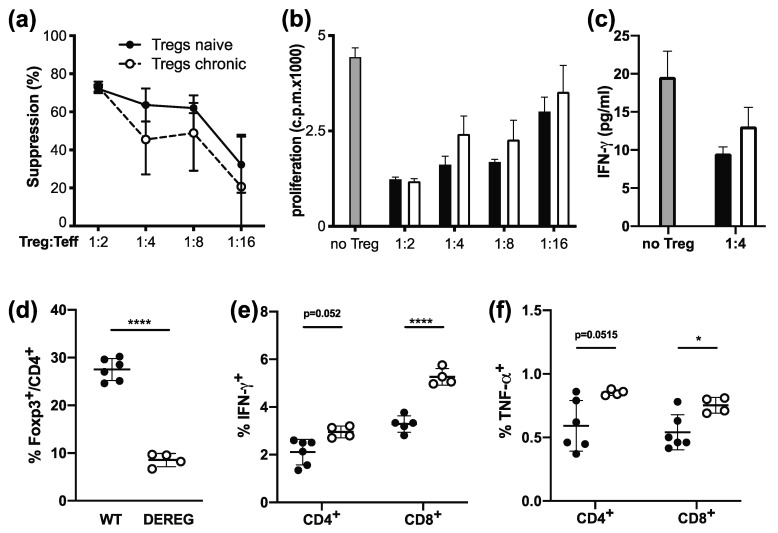
Tregs maintain suppressive function during chronic infection. (**a**–**c**) CD4^+^GFP^+^ Tregs were sorted from naive or day 30–40 LCMV Clone 13-infected Foxp3-GFP.KI reporter mice and co-cultured with CD4^+^GFP^-^ Th1 effector cells (from LCMV WE-infected mice) stimulated with anti-CD3 (1 µg/mL) and irradiated splenic APCs. After 2 days, proliferation was determined by [^3^H]-thymidine incorporation. Treg-mediated suppression of proliferation (**a**), proliferation (**b**), and IFN-γ concentrations in the culture supernatant (**c**) are displayed (mean ± SD; biological replicates, *n* = 3; 1 representative experiment of 3 is shown). (**d**–**f**) DEREG and WT control mice were infected with LCMV Clone 13 and injected with diphtheria toxin on day 10, 12, 14, and 16 post infection to deplete Tregs or as a control. On day 17, spleens were harvested, Treg frequencies were determined by flow cytometry (**d**), and frequencies of LCMV-specific T cells were determined in splenocytes by re-stimulation with LCMV gp33 and gp61 followed by intracellular cytokine staining and flow cytometric analysis of IFN-γ (**e**) and TNF-α (**f**) production by CD8^+^ or CD4^+^ T cells (mean ± SD; *n* = 5–6 (WT) or 4 (DEREG); 1 representative experiment out of 2 is shown). * *p* < 0.05, **** *p* < 0.0001; *t*-Test.

Tregs have been shown to contribute to pathogen persistence by inhibiting effector T cell function [5]. Nevertheless, as effector T cells acquire a dysfunctional phenotype during chronic LCMV infection [27], we first wanted to confirm that Tregs retain their suppressive function during chronic infection with LCMV Clone 13. To address this, we compared the ability of Tregs isolated from naive or chronically LCMV infected mice to suppress CD4^+^ Th1 effector cells in an in vitro suppression assay. CD4^+^GFP^+^ Tregs from Foxp3-GFP.KI reporter mice were sorted from naive or chronically LCMV infected mice (day 30) and co-cultured with CD4^+^GFP^-^ Th1 effector cells isolated from LCMV-infected mice and stimulated with anti-CD3. We observed no differences in the ability of Tregs from naive or chronically infected mice to suppress Th1 effector T cell proliferation or their secretion of IFN-γ (Figure 1a–c). The in vitro suppressive capacity of Tregs is thus unimpaired during chronic LCMV infection.

To test their functionality in vivo, we took advantage of DEREG mice, which express the diphtheria toxin receptor under the control of the Foxp3 promotor and thus allow for deletion of Tregs through diphtheria toxin administration [28]. DEREG mice were infected with LCMV Clone 13 and Tregs depleted starting from day 10 post infection, when chronic infection is already established. In line with previous studies [29], DTR^-^ Tregs had re-emerged in DEREG mice 7 days after the beginning of Treg depletion, but Treg numbers remained significantly reduced compared to control animals (Figure 1d). On day 17 post infection (7 days after the beginning of Treg depletion), the effector T cell response was analyzed, and despite the re-emerging Treg population in DEREG mice (Figure 1d), a slight increase in the frequency of IFN-γ^+^ and TNF-α^+^ CD8^+^ and CD4^+^ effector T cells could be observed following Treg depletion (Figure 1e,f). These results confirm that Tregs are functional in chronic viral infections and dampen effector T cell responses.

### 2.2. Tregs Undergo Classical Th1-Specific Specialization in Persistent Infection

During acute Th1 responses, Tregs undergo functional specialization, which is characterized by the co-expression of the Th1 master transcription factor T-bet with Foxp3 as well as expression of the chemokine receptor CXCR3 [9,12]. However, whether a similar specialization into type 1 Tregs takes place during persistent infections has not been addressed. As Tregs isolated from chronically infected mice were functional both in vitro and in vivo, we next investigated whether they also undergo functional specialization into type 1 Tregs during persistent viral infections. To obtain a more comprehensive picture of phenotypic changes in Tregs during persistent Th1-dominated infections, we not only analyzed Tregs during chronic LCMV Clone 13 infection but also investigated Tregs following MCMV infection, which induces a latent infection. Furthermore, sublingual infections with *Candida albicans* strain 101 served as a persistent but Th17-polarized fungal control infection [30].

**Figure 2:**
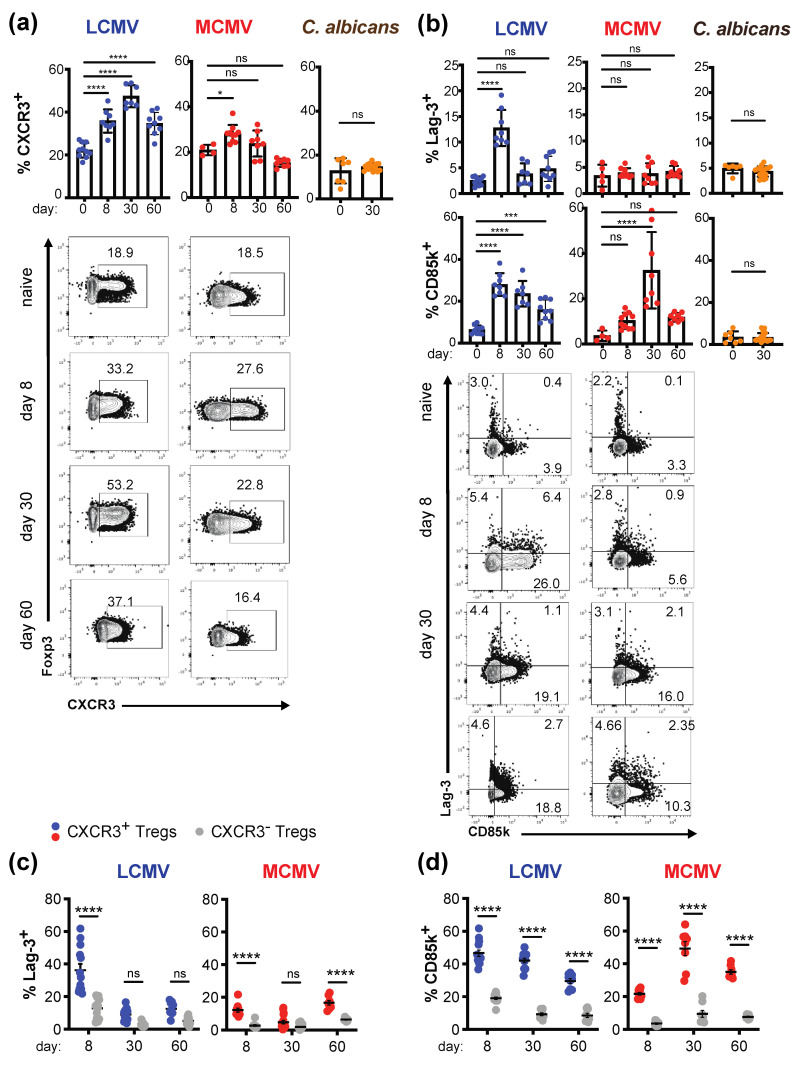
Tregs undergo Th1-specific specialization in persistent viral infection infections. C57BL/6 mice were left naïve (day 0) or infected with LCMV Clone 13 (blue, 2 × 10^6^ ffu i.v.), MCMV (red, 2 × 10^5^ pfu i.v.), or *C. albicans* strain 101 (orange, 2.5 × 10^6^ cfu sublingual), and sacrificed 8, 30, and 60 days post infection. Spleens (LCMV, MCMV) or cervical lymph nodes (*C. albicans*) were harvested and directly analyzed ex vivo by flow cytometry. (**a**,**b**) Representative plots (left) and summary graphs (right) for frequencies of CXCR3^+^ (**a**), Lag-3^+^, and CD85k^+^ (**b**) Foxp3^+^CD4^+^ Tregs are depicted. (**c**,**d**) Frequencies of Lag-3^+^ (**c**) or CD85k^+^ (**d**) cells among CXCR3^+^ (red) or CXCR3^-^ (grey) Tregs were also determined in LCMV- and MCMV-infected mice (mean ± SD; biological replicates: naive *n* = 5–7; LCMV *n* = 8–12, MCMV *n* = 8–10, *C. albicans n* = 19; pooled data of 2–4 independent experiments). * *p* < 0.05, **** *p* < 0.0001, ns: not significant; for comparison of two or more groups, 2-sided *t*-Test or one-way ANOVA was used, respectively.

Despite a transient decrease of PD-1 upon MCMV infection, Tregs from both LCMV- and MCMV-infected mice showed robust expression of the co-inhibitory receptors CTLA-4, PD-1, and TIGIT (Figure S1), which are associated with Treg suppressive function [31,32,33]. Furthermore, both viral but not fungal *C. albicans* infections resulted in an increase of CXCR3^+^ Tregs (Figure 2a), suggesting that a similar type 1 specialization process as has been reported in acute infections takes place in persistent infections and may be maintained in chronic LCMV infection. Although, the frequency of CXCR3^+^ Tregs starts to decrease 60 days after LCMV infection as, at this point, the virus starts to get cleared due to the emerging antibody response [34,35]. We next assessed the expression of the co-inhibitory receptors Lag-3 and CD85k, which are specifically induced in the context of acute type 1 responses [15]. Lag-3, which peaks early in acute Th1 infections [15], was only transiently upregulated in mice persistently infected with LCMV but not MCMV (Figure 2b). In contrast, the frequency of CD85k^+^ Tregs was robustly induced in both persistent viral infections (Figure 2b). While expression was maintained in LCMV infection, CD85k was only transiently induced during MCMV infection, and during the latent phase (day 60) only a trend towards higher CD85k expression could be observed, which did not reach significance. Importantly, persistent *C. albicans* infection, which is marked by a Th17 response [36,37], did not induce Lag-3 or CD85k expression in Tregs (Figure 2b), confirming the specificity of Lag-3 and CD85k as Th1-specific co-inhibitory receptors on Tregs. This was further supported by the fact that Lag-3 and CD85k were almost exclusively expressed in CXCR3^+^ Tregs (Figure 2c,d). Tregs thus undergo specialization into type 1 Tregs during persistent infection, which is marked by expression of CXCR3 and the Th1-specific co-inhibitory receptor CD85k and is maintained throughout the course of chronic LCMV but not latent MCMV infection, suggesting that active viral replication is necessary to maintain increased levels of CD85k^+^ type 1 Tregs.

### 2.3. Effector T Cells Express the CD85k Ligand ALCAM during Chronic Infection

**Figure 3:**
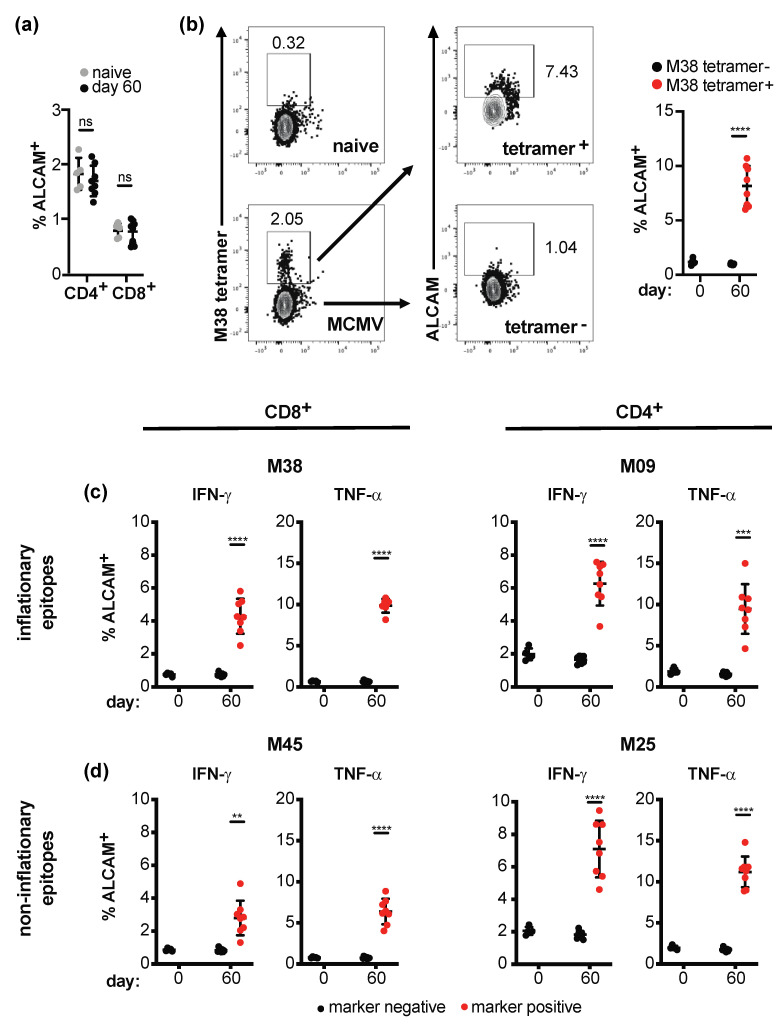
ALCAM is expressed on MCMV-specific effector T cells. C57BL/6 mice were left naïve (day 0) or infected with MCMV (2 × 10^5^ pfu i.v.). 60 days post infection, spleens were harvested and frequencies of ALCAM^+^ total CD4^+^ or CD8^+^ T cells (**a**) and ALCAM expression within CD8^+^ T cells stained by the M38-tetramer (**b**) were determined by flow cytometry. (**c**,**d**) Samples were re-stimulated with peptides of inflationary (**c**, M38 and M09) or non-inflationary (**d**, M45 and M25) MCMV epitopes for 4 h before staining for ALCAM in conjunction with intracellular cytokines among CD8^+^ or CD4^+^ T cells. Summary data (**a**–**d**) and representative stainings (**b**) are displayed (mean ± SD; biological replicates: naive *n* = 4, day 60 *n* = 8). ** *p* < 0.01, *** *p* < 0.001, **** *p* < 0.0001, ns: not significant; for comparison of two or more groups, 2-sided *t*-Test or one-way ANOVA was used, respectively.

Tregs expressing CD85k are highly suppressive for Th1 effector cells [15]. Given that CD85k^+^ Tregs are induced and in the case of chronic LCMV infection even maintained during viral infection, we hypothesized that the interaction of CD85k on Tregs with its ligand ALCAM [19] on effector cells could limit the effector T cell response during persistent infection. To test this hypothesis, we first analyzed ALCAM expression on effector cells during the latent phase of MCMV infection (day 60). Although we did not observe an overall increase in ALCAM expression on effector T cells in MCMV-infected animals (Figure 3a), MCMV-specific T cells (identified by tetramer staining or re-stimulation with MCMV peptides) expressed higher levels of ALCAM than naïve, tetramer-, or cytokine-negative T cells (Figure 3b–d). However, the increase in ALCAM was only modest and comparable in T cells specific for inflationary and non-inflationary epitopes.

**Figure 4:**
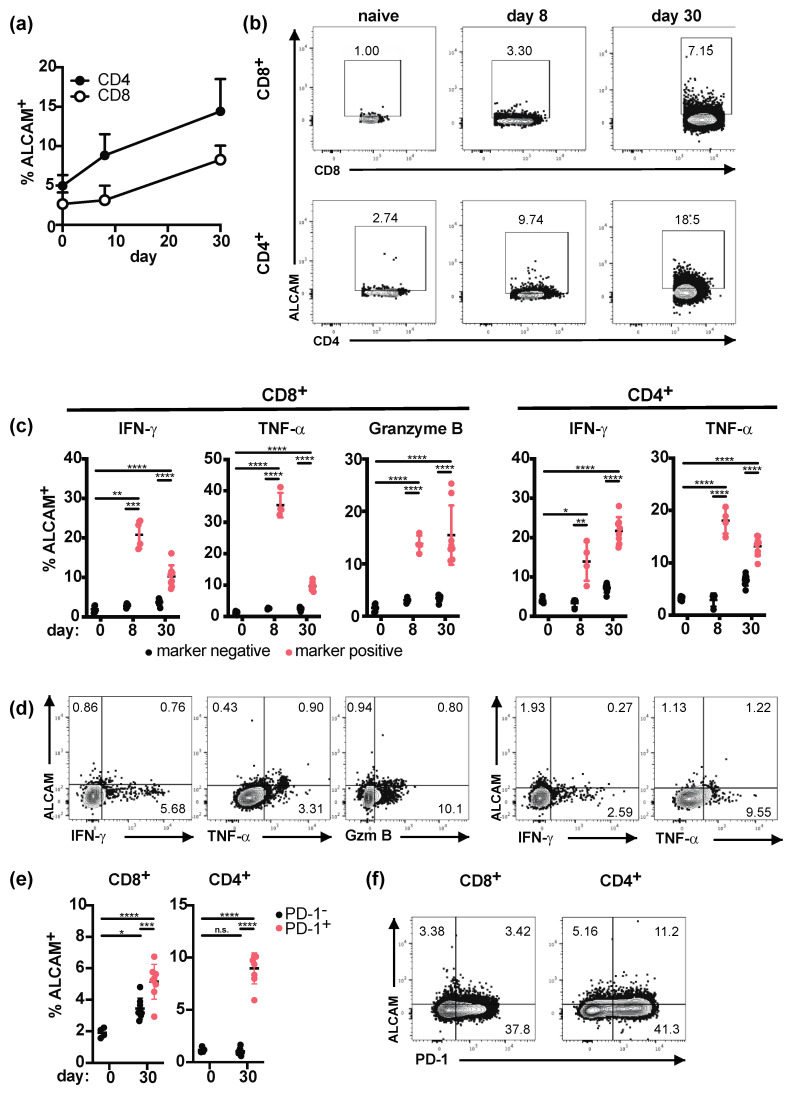
ALCAM is expressed on LCMV-specific effector T cells. C57BL/6 mice were left naïve (day 0) or infected with LCMV Clone 13 (2 × 10^6^ ffu i.v.). Then, 8 or 30 days post infection, spleens were harvested, and frequencies of ALCAM^+^ total CD4^+^ or CD8^+^ T cells were determined by flow cytometry (**a**,**b**). Samples were re-stimulated with LCMV gp33 and gp61 for 4 h before staining for ALCAM in conjunction with intracellular cytokines and granzyme (**c**,**d**) or PD-1 (**e**,**f**) among CD8^+^ or CD4^+^ T cells. Summary data (**a**,**c**,**e**) and representative staining (**b**,**d**: day 8, **f**: day 30) are displayed (mean ± SD; biological replicates: naive *n* = 4–6, day 8 *n* = 4, day 30 *n* = 8, pooled from in 2–3 independent experiments). * *p* < 0.05, ** *p* < 0.01, *** *p* < 0.001, **** *p* < 0.0001, ns: not significant; for comparison of multiple groups, two-way ANOVA with multiple comparisons was used.

Next, we analyzed ALCAM expression during chronic LCMV infection, where we had observed a sustained increase in CD85k^+^ type 1 Tregs (Figure 2b). WT B6 mice were infected with LCMV clone 13, and ALCAM expression on CD4^+^ and CD8^+^ T cells was analyzed during the acute (day 8) and chronic (day 30) phase of the infection and compared to naïve mice (day 0). We could indeed observe a steady increase of ALCAM expression in both T cell populations that was maintained throughout the course of infection (Figure 4a,b). Furthermore, LCMV-specific re-stimulation of T cells with the immunodominant peptides gp33 (for CD8^+^ T cells) and gp61 (for CD4^+^ T cells) revealed that the frequency of ALCAM^+^ cells was higher in LCMV-specific effector T cells producing IFN-γ, TNF-α, or granzyme B (Figure 4c,d).

LCMV-specific effector T cells become exhausted during the later stages of infection and show impaired cytokine production upon re-stimulation but high expression of the co-inhibitory receptor PD-1 [38]. We thus also analyzed ALCAM expression on PD-1^+^ effector T cells at the chronic stage of infection (day 30) and found a significantly higher proportion of ALCAM^+^ cells within PD-1^+^ effector T cells compared to PD-1^-^ controls or naïve T cells (Figure 4e). ALCAM is thus induced on LCMV-specific effector T cells during chronic LCMV infection and its expression is maintained on exhausted T cells.

### 2.4. Type 1 Tregs Limit ALCAM-Expressing Effector T Cells

**Figure 5:**
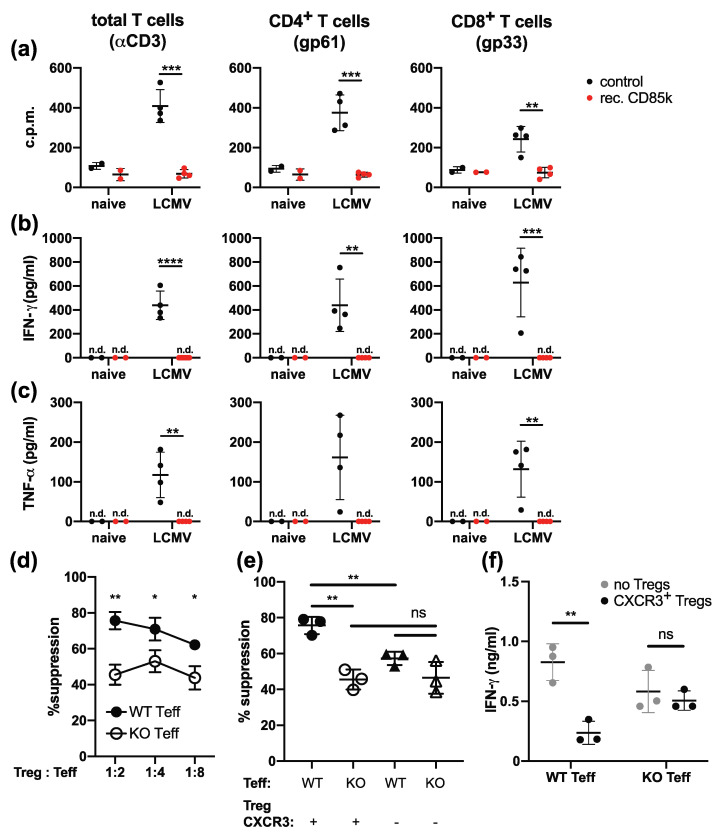
Suppression of effector T cells from chronic infection. (**a**–**c**) C57BL/6 mice were left naïve (day 0) or infected with LCMV Clone 13 (2 × 10^6^ ffu i.v.). Then, 8 days post infection, spleens were harvested and cells were re-stimulated with anti-CD3 (**left**), LCMV gp61 (**middle**), or gp33 (**right**) in the presence (red) or absence (black) of recombinant CD85k. After 2 days, proliferation was determined by [^3^H]-thymidine incorporation (**a**) and the secretion of IFN-γ (**b**) and TNF-α (**c**) into the culture supernatant was determined by CBA (mean ± SD; *n* = 4; 1 representative experiment of 2 is shown). (**d**–**f**) CXCR3^+^CD4^+^GFP^+^ and CXCR3^-^CD4^+^GFP^+^ Tregs were sorted from LCMV Clone 13-infected Foxp3-GFP.KI reporter mice (day 25) and co-cultured with CD4^+^ Th1 effector cells sorted from C57BL/6 WT mice (filled symbols) or ALCAM^-/-^ mice (open symbols) also infected with LCMV Clone 13 (day 10). Cells were stimulated with anti-CD3 and irradiated splenic APCs for 2 days before proliferation was determined by [^3^H]-thymidine incorporation. Treg-mediated suppression of proliferation (**d**,**e**) and IFN-γ concentrations in the culture supernatant (**f**) are shown (mean ± SD; *n* = 3; 1 representative experiment of 2 is shown). * *p* < 0.05, ** *p* < 0.01, *** *p* < 0.001, **** *p* < 0.0001, ns: not significant; for comparison of two or more groups, 2-sided *t*-Test or one-way ANOVA were used, respectively.

CD85k expressed on tolerogenic DCs can inhibit T cell proliferation through binding to its ligand ALCAM [19]. Based on the upregulation of ALCAM on effector cells during chronic LCMV infection (Figure 4), we next tested whether CD85k expressed on type 1 Tregs could suppress effector T cells during chronic infection. To this end, we infected WT B6 mice with LCMV Clone 13 to generate ALCAM-expressing effector T cells. On day 8 post infection, splenocytes were isolated and re-stimulated with the immunodominant LCMV peptides (gp33 and gp61) or with anti-CD3 with or without the addition of recombinant CD85k. After 2 days of stimulation, proliferation was determined by ^3^H-Thymidine incorporation and cytokine secretion into the supernatant was assessed by cytometric bead array (Figure 5a–c). Indeed, recombinant CD85k strongly suppressed proliferation of CD4^+^ as well as CD8^+^ T cells, confirming that it can inhibit effector T cell expansion during chronic viral infection (Figure 5a). Furthermore, secretion of IFN-γ and TNF-α was also strongly impaired in the presence of recombinant CD85k (Figure 5b,c). CD85k is thus able to potently suppress the expansion and function of effector T cells arising during chronic LCMV infection.

Finally, we tested whether the CD85k-expressing type 1 Tregs induced upon chronic LCMV infection suppress effector T cells by engaging ALCAM on these cells. We performed an in vitro suppression assay to determine the ability of type 1 Tregs from chronic mice to suppress effector T cells from chronically infected WT or ALCAM KO mice. CXCR3^+^CD4^+^GFP^+^ type 1 Tregs were sorted from Foxp3-GFP.KI reporter mice during the chronic phase of LCMV infection (day 25) and co-cultured with CD4^+^ effector T cells from either WT or ALCAM KO mice also infected with LCMV clone 13 and stimulated with anti-CD3. In line with our hypothesis, we observed reduced suppression of ALCAM KO effector T cells by CXCR3^+^ type 1 Tregs (Figure 5d). CXCR3^+^ type 1 Tregs also did not exert enhanced suppression towards ALCAM KO Th1 cells when compared to CXCR3^-^ Tregs as they did towards WT Th1 cells (Figure 5e and Figure S2). In addition, CXCR3^+^ type 1 Tregs could not suppress the secretion of IFN-γ by ALCAM KO T cells, while suppression of IFN-γ secretion was observed in WT Th1 cells (Figure 5f). Taken together, these data show that type 1 Tregs arising during chronic viral infection suppress effector Th1 cells by engaging ALCAM.

## 3. Discussion

In this study, we showed that Tregs undergo functional specialization into type 1 Tregs during persistent viral infection and identified CD85k as a co-inhibitory receptor with sustained expression in this Treg subset during chronic infection. The CD85k ligand ALCAM is induced and maintained on virus-specific effector T cells during chronic LCMV infection and recombinant CD85k can inhibit LCMV-specific effector T cells. Most importantly, type 1 Tregs arising during chronic LCMV infection, which specifically express CD85k, suppress Th1 effector cells in an ALCAM-dependent manner.

Th1 responses elicited upon acute infectious challenge induce the specialization of Tregs into T-bet-expressing CXCR3^+^ type 1 Tregs [9,12]. While expression of T-bet in inflammation-experienced Treg was shown to be highly stable over time [12], this study only examined Tregs induced in acute infections, where immune activation and the presence of polarizing cytokines are transient. We show here that type 1 specialization of Tregs also takes place in persistent viral infections. Although the specialization into type 1 Tregs could be observed upon both LCMV and MCMV infection, the induction of CXCR3^+^ Tregs was more pronounced in chronic LCMV infection. These differences may be due to the nature of the infections, which are characterized by high and sustained antigen loads in LCMV Clone 13 infection [27] or latency with undetectable virus in the spleen and sporadic re-activation in persistent MCMV infection [39]. Furthermore, potentially higher concentrations of the polarizing cytokines IFN-γ or IL-27 could induce a more pronounced Treg specialization upon LCMV infection. Nevertheless, the frequency of CD85k^+^ Tregs was clearly increased in the later phase of both infectious settings, albeit it was only maintained in chronic and not latent infection. This suggests that active viral replication and inflammation are necessary to maintain increased levels of CD85k^+^ type 1 Tregs. As such, it will be interesting to determine whether, like CXCR3, CD85k expression is also induced by IFN-γ and IL-27 and the transcription factor T-bet or whether additional factors contribute to the induction of CD85k.

Treg specialization has been reported in the context of type 1, type 2, and type 17 responses and is marked by the mirrored expression of transcription factors and chemokine receptors in effector and regulatory T cells [9,10,11]. The expression of corresponding chemokine receptors allows specialized Tregs to migrate to the same site as the respective effector T cells and is therefore important for effective immune suppression [9,12]. Nevertheless, how or whether the transcriptional program induced by the lineage-specific transcription factors may contribute to enhanced or even selective suppressive function is still largely unclear. Interestingly, the induced deletion of T-bet expression in Tregs had no immediate effects on overall immune homeostasis, while the ablation of T-bet-expressing Tregs resulted in a loss of control of Th1 responses and autoimmunity [12]. This suggests that once the Tregs are properly positioned, T-bet (and CXCR3) are no longer required for efficient Treg function. Nevertheless, type 1 Tregs induced during acute infections are more suppressive towards Th1 cells than CXCR3^-^ or naïve Tregs in in vitro suppression assays, where obviously migration does not play a role [15]. Importantly, this enhanced suppression was only observed towards Th1 but not naïve CD4^+^ T cells and thus was not a result of overall enhanced function but of functional specialization of type 1 Tregs [15]. We have previously reported that Th1-specific co-inhibitory receptors on Tregs, including CD85k, are predictive of enhanced Th1 suppression [15]. Here, we further extended this finding and functionally link expression of the CD85k ligand ALCAM with the ability of Th1 cells to be efficiently suppressed by type 1 Tregs in the setting of a chronic viral infection.

Interestingly, CD85k is prominently expressed in Tregs that lack the kinase CK2β and are unable to control Th2 immune responses [20]. The inability of CD85k^+^ Tregs to suppress Th2 responses in CK2β-deficient mice further supports a functional contribution of CD85k in the specific suppression of type 1 immunity. A number of co-inhibitory receptors, including CTLA-4, Lag-3, and TIGIT, have been shown to contribute to overall Treg suppressive function [31,32,40]. The results we present here add CD85k to the list of co-inhibitory receptors that contribute to specific Treg-mediated suppression and suggest it fulfils a context-specific inhibitory role in that it is specifically induced to inhibit type 1 immune responses by binding to its ligand ALCAM on effector T cells.

## 4. Materials and Methods

### 4.1. Mice, Pathogens, and Infections

All animal experiments were reviewed and approved by the cantonal veterinary office of Zurich (licenses ZH119/17, ZH178/17, ZH179/17, ZH115/17, ZH114/17, ZH183/15, ZH058/20, and ZH166/18) and performed in accordance with Swiss legislation. Mice were bred and housed at the Laboratory Animal Sciences Center (LASC) Zurich, Switzerland or the ETH Phenomics Center Zurich, Switzerland. C57BL/6Rj (WT) mice were purchased from Janvier Laboratories (Saint Berthevin Cedex, France). Foxp3-GFP.KI reporter mice [41], DEREG mice [28], and ALCAM^−/−^ mice [42] were described previously.

LCMV Clone 13 was propagated on BHK21 cells. MCMV (BAC-derived Smith strain on a MCK-2-repaired background [43]) was propagated on M2-10B4 cells. *C. albicans* strain 101 was grown overnight in YPD medium for 15–18 h at 30 °C and 180 rpm before infection. Sex- and age-matched mice, 6–12 weeks of age, were infected i.v. with 2 × 10^6^ ffu LCMV Clone 13, or 2 × 10^5^ pfu MCMV, or sublingually with 2.5 × 10^6^ *C. albicans* yeast cells as described previously [36]. To deplete Tregs, 200 ng of diphtheria toxin (Merck, Buchs, Switzerland) in PBS were injected i.p. on days 10, 12, 14, and 16 of the experiment.

### 4.2. Isolation of Leukocytes

For analysis, spleens (from LCMV Clone 13 and MCMV infections) and draining cervical lymph nodes (*C. albicans* strain 101 infections) were collected and dissociated by carefully pushing them through a fine metal mesh or 70-μm cell strainer, respectively. Red blood cell lysis was performed for spleen samples using ACK buffer (155 mM NH_4_Cl, 1 mM KHCO_3_, 0.1 mM NA_2_EDTA in ddH_2_O, pH 7.2–7.4) prior to staining for flow cytometry.

### 4.3. Flow Cytometry

Single-cell suspensions were surface stained with fluorophore-conjugates antibodies for 20 min, followed by fixation/permeabilization for 45 min using the Foxp3 Staining Buffer Set (eBioscience, Waltham, MA, USA) for transcription factor staining or 10 min using the BD Fixation/Permeabilization Solution kit (BD Bioscience, Franklin Lakes, NJ, USA) for intracellular cytokine staining, followed by intracellular staining for 30–40 min at room temperature. For intracellular cytokine staining, isolated bulk splenocytes were re-stimulated using αCD3 (2 μg/mL, 145-2C11, BioXcell, Lebanon, NH, USA), the immunodominant LCMV peptides gp61 and gp33 (1 μg/mL; gp61: GLKGPDIYKGVYQFKSVEFD; gp33: KAVYNFATM), or inflationary (M09, M38) and non-inflationary (M25, M45) MCMV peptides (1 μg/mL; M09: GYLYIYPSAGNSFD; M25: NHLYETPISATAMVI; M38: SSPPMFRV; M45: HGIRNASFI) for 4 h at 37 °C in the presence of Brefeldin A (5 μg/mL, Biolegend, London, UK). Fluorophore-conjugated antibodies against murine CD4 (RM4-5 or GK1.5), CD8 (53-6.7), Foxp3 (FJK-16s), CXCR3 (CXCR3-173), CD85k (H1.1), LAG-3 (C9B7W), TIGIT (1G9), PD-1 (J43), CTLA-4 (UC10-4B9), IFN-γ (XMG1.2), TNF-α (MP6-XT22), GranzymeB (GB12), and ALCAM (eBioALC48) were purchased from Biolegend, eBioscience, or R&D Systems (Abingdon, UK). MHC Class I tetramers for M38 (K^b^ restricted) were produced as described [44], tetramerized by PE-labelled streptavidin, and added during the extracellular staining step. The LIVE/DEAD Fixable Near-IR Dead Cell Stain Kit (Invitrogen) was used to exclude dead cells. Counting beads (CountBright, Invitrogen, Waltham, MA, USA) were added before flow cytometric acquisition to determine absolute cell numbers. Data were acquired on a BD LSRFortessa or BD FACSCanto II cytometer (BD Biosciences, Franklin Lakes, NJ, USA) and analyzed using Flowjo Software (Flowjo, LLC, Ashland, OR, USA). FACS gates were drawn based on isotype control-stained samples for each experimental group.

### 4.4. In Vitro Co-Culture and Treg Suppression Assays

For co-culture assays, splenocytes were stimulated in 96-well plates at 5 × 10^5^ cells/well with anti-CD3 (1 μg/mL, 145-2C11) or the immunodominant LCMV peptides gp61 and gp33 (1 mg/mL) for 2 days at 37 °C. Where indicated, recombinant CD85k (12.5 μg/mL, Cloud Clone Corp.) or Tregs were added to the culture. After 2 days, 1 µCi [^3^H]-thymidine (PerkinElmer) was added per well for an additional 18–22 h before harvest and quantification of [^3^H]-thymidine incorporation.

Suppression assays were performed as described previously [15]. In brief, CD4^+^ T cells were enriched from naïve or LCMV-infected (day 25–40, chronic phase) Foxp3-GFP.KI mice from spleen and peripheral lymph nodes (inguinal, brachial, axillary, cervical) using anti-CD4-positive selection beads (Mojo, BioLegend). Tregs were then FACS sorted as CD4^+^GFP^+^ cells on a FACSAria III (BD Biosciences). Th1 effector cells were FACS sorted from spleen and lymph nodes of day 10–12 LCMV WE or LCMV Clone 13-infected Foxp3-GFP.KI mice as CD4^+^GFP^-^ cells, respectively. CD4^+^GFP^-^ effector cells (4 × 10^4^ cells/well) and CD4^+^GFP^+^ Treg cells were co-cultured in triplicates at effector T: Treg cell ratios of 2:1, 4:1, 8:1, and 16:1 in the presence of soluble anti-CD3 (1 µg/mL, 145-2C11) and irradiated splenic antigen-presenting cells (APCs) (2 × 10^5^/well) at 37 °C. Assays were performed in DMEM (10% FCS, β-mercaptoethanol (50 mM), sodium pyruvate (1 mM, Gibco, Waltham, MA, USA), non-essential amino acids (Gibco), MEM vitamins (Gibco), penicillin (50 U/mL, Gibco), streptomycin (50 µg/mL, Gibco), and glutamine (2 mM, Gibco)). After 48 h, 1 µCi [^3^H]-thymidine (PerkinElmer, Schwerzenbach, Switzerland) was added to each well followed by 18–22 h of additional incubation. Cells were then harvested and [^3^H]-thymidine incorporation quantified. Percent suppression = ([mean value of c.p.m. of CD4^+^Foxp3^−^ controls]—[c.p.m. of wells effector T and Treg cells]/[mean c.p.m. of CD4^+^Foxp3^-^ controls]) × 100.

### 4.5. Statistical Analysis

Statistical significance was assessed using GraphPad Prism version 8 (GraphPad Software, San Diego, CA, USA) and Gaussian distribution was assumed for all datasets. Differences between individual groups were determined using the two-sided t Test or between more than two groups using one- or two-way ANOVA with multiple comparisons. Outlier calculation was performed using the ROUT method. Statistical significance values are indicated as follows: *p* < 0.05 (*), *p* < 0.01 (**), *p* < 0.001 (***), and *p* < 0.0001 (****).

## Data Availability

The data presented in this study are available on request from the corresponding author.

## Supplementary Materials

Supplementary Materials can be found at https://www.mdpi.com/1422-0067/22/1/31/s1. Figure S1: Treg characterization during chronic infection. Figure S2: Suppression by type 1 Tregs is ALCAM-dependent.

## Abbreviations

Treg	regulatory T cell
LCMV	Lymphocytic choriomeningitis virus
MCMV	Murine cytomegalovirus
APC	antigen presenting cell
