# Supplementary material for: CD85k Contributes to Regulatory T Cell Function in Chronic Viral Infections
Source: Int J Mol Sci. 2020 Dec 22;22(1):31. doi: 10.3390/ijms22010031 (PMC7792974; doi:10.3390/ijms22010031)
Supplement: Supplementary file 1 [file ijms-22-00031-s001.pdf]

International Journal of *Molecular Sciences*

*Supplementary Material*

# CD85k contributes to regulatory T cell function in chronic viral infections

**Anna Estrada Brull [1,†], Felix Rost [1,†], Josua Oderbolz [2], Florian R. Kirchner [1,3], Salomé Leibundgut-Landmann [1,3], Annette Oxenius [2] and Nicole Joller [1,*]**

[1] University of Zurich, Institute of Experimental Immunology, 8057 Zurich, Switzerland; anna.estradabrull@uzh.ch (A.E.B.); rost@immunology.uzh.ch (F.R.); florian.kirchner@uzh.ch (F.R.K.); salome.leibundgut-landmann@uzh.ch (S.L.-L.)
[2] ETH Zurich, Institute of Microbiology, 8093 Zurich, Switzerland; josua.oderbolz@micro.biol.ethz.ch (J.O.); aoxenius@micro.biol.ethz.ch (A.O.)
[3] University of Zurich, Section of Immunology, Vetsuisse Faculty, 8057 Zurich, Switzerland
* Correspondence: nicole.joller@uzh.ch
† These authors contributed equally.

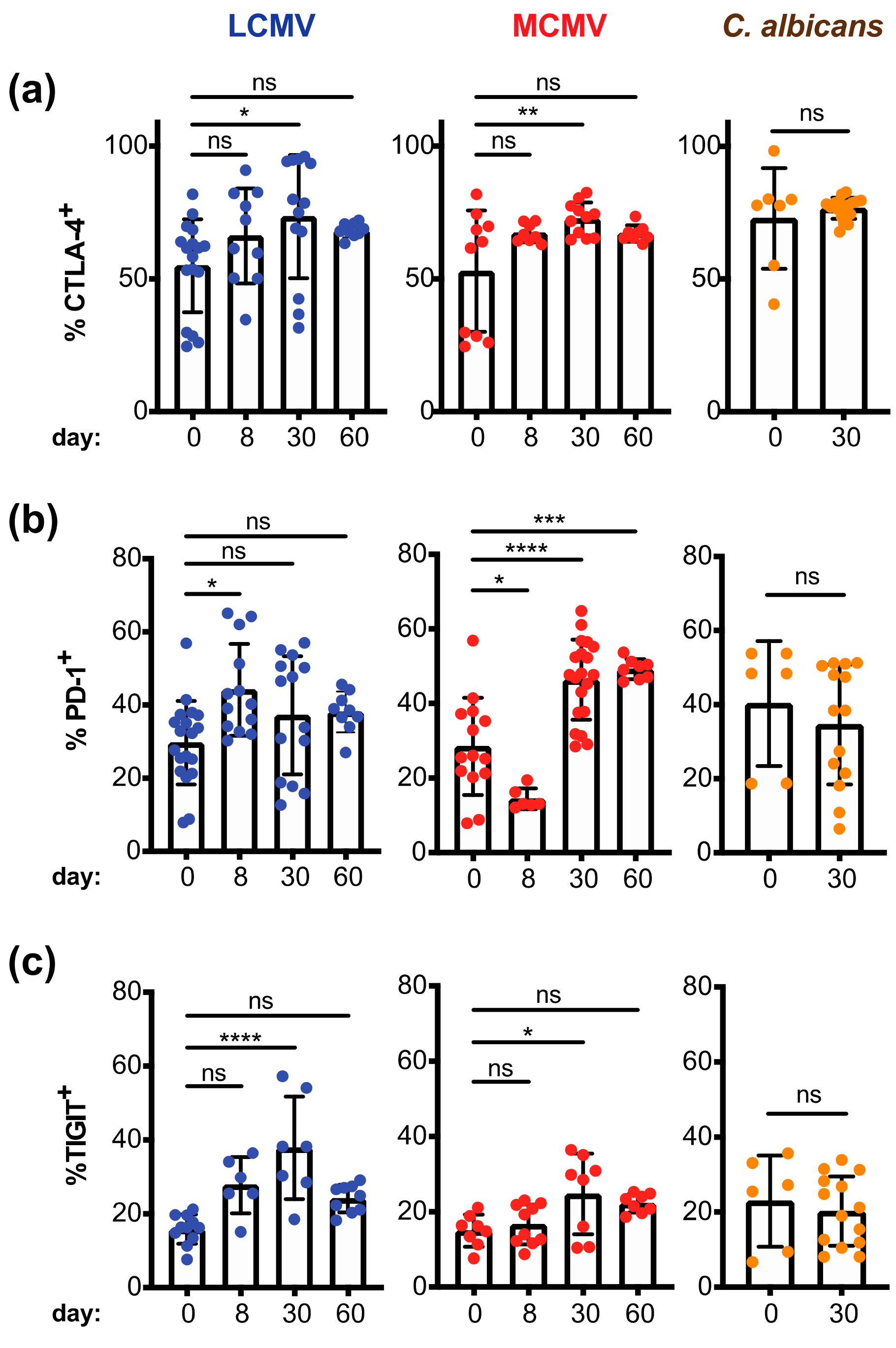


**Figure S1.** Treg characterization during chronic infection. C57BL/6 mice were infected with LCMV Clone 13 (blue, 2x10$^6$ ffu i.v.), MCMV (red, 2x10$^5$ ffu i.v.), or *C. albicans* strain 101 (orange, 2.5x10$^6$ cfu sublingual), sacrificed 8, 30, and 60 days post infection. Spleens (LCMV, MCMV) or cervical lymph nodes (*C. albicans*) were harvested and directly analyzed *ex vivo* by flow cytometry. Summary graphs for frequencies of CTLA-4$^+$ (**a**), PD-1$^+$ (**b**), and TIGIT$^+$ (**c**) Tregs are depicted (Mean ± SEM; biological replicates: LCMV / MCMV / *C. albicans*: Naive n = 9-14 / 9-15 / 7; day 8 n = 8-13 / 4-10; day 30 n = 7-13 / 5-20 / 19, analyzed in 6 / 2-4 / 2-4 independent experiments). *p < 0.05, **p < 0.01, ***p < 0.001, ****p < 0.0001; for comparison of two, three or six groups 2-sided t-Test, one-way ANOVA, or two-way ANOVA with multiple comparisons were used, respectively.

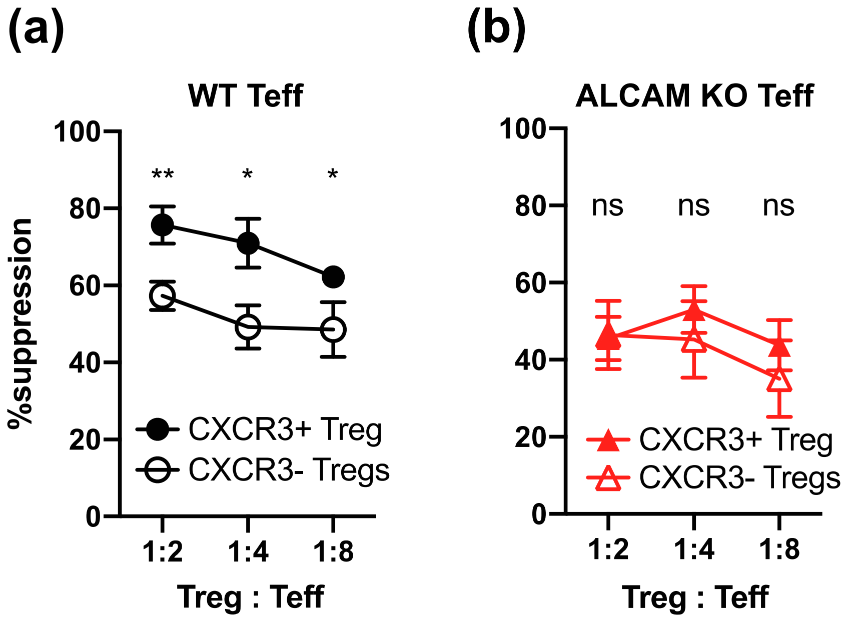


**Figure S2.** Suppression by type 1 Tregs is ALCAM-dependent. $CXCR3^+CD4^+GFP^+$ and $CXCR3^-CD4^+GFP^+$ Tregs were sorted from LCMV Clone 13 infected Foxp3-GFP reporter mice (day 25) and co-cultured with $CD4^+$ Th1 effector cells sorted from C57BL/6 WT mice (**a**) or $ALCAM^{-/-}$ mice (**b**) also infected with LCMV Clone 13 (day 10). Cells were stimulated with anti-CD3 and irradiated splenic APCs for 2 days before proliferation was determined by [$^3$H]-thymidine incorporation and Treg-mediated suppression was calculated (mean ± SD; n = 3; 1 representative experiment of 2 is shown). $^*p < 0.05$, $^{**}p < 0.01$; t-Test.

© 2020 by the authors. Licensee MDPI, Basel, Switzerland. This article is an open access article distributed under the terms and conditions of the Creative Commons Attribution (CC BY) license (http://creativecommons.org/licenses/by/4.0/).
